# Implication of the PI3K/Akt/mTOR Pathway in the Process of Incompetent Valves in Patients with Chronic Venous Insufficiency and the Relationship with Aging

**DOI:** 10.1155/2018/1495170

**Published:** 2018-07-02

**Authors:** Miguel A. Ortega, Ángel Asúnsolo, Javier Leal, Beatriz Romero, María J. Alvarez-Rocha, Felipe Sainz, Melchor Álvarez-Mon, Julia Buján, Natalio García-Honduvilla

**Affiliations:** ^1^Department of Medicine and Medical Specialities, Faculty of Medicine and Health Sciences, University of Alcalá, Alcalá de Henares, Spain; ^2^Networking Biomedical Research Center on Bioengineering, Biomaterials and Nanomedicine (CIBER-BBN), Alcalá de Henares, Spain; ^3^Ramón y Cajal Institute of Sanitary Research (IRYCIS), Madrid, Spain; ^4^Department of Surgery, Medical and Social Sciences, Faculty of Medicine and Health Sciences, University of Alcalá, Alcalá de Henares, Madrid, Spain; ^5^Angiology and Vascular Surgery Service, Ruber International Hospital, Madrid, Spain; ^6^Angiology and Vascular Surgery Service, Central University Hospital of Defense-UAH, Madrid, Spain; ^7^Immune System Diseases-Rheumatology and Oncology Service, University Hospital Príncipe de Asturias, Alcalá de Henares, Madrid, Spain; ^8^University Center of Defense of Madrid (CUD-ACD), Madrid, Spain

## Abstract

Chronic venous insufficiency (CVI) is a multifactorial disease, commonly caused by valvular incompetence (clinically diagnosed by venous reflux) and venous hypertension. The incidence of these factors clearly increases with patient age, and aging is one of the risk factors involved. The activity of the PI3K/Akt/mTOR pathway is considered fundamental in vascular pathologies, and understanding its involvement would help in the development of possible therapeutic targets. This is an observational, analytical, and prospective cohort study that reviewed 110 patients with CVI scheduled to undergo stratified saphenectomy. They were distributed according to the presence (R = 81) or absence (NR = 29) of valvular incompetence (venous reflux) diagnosed clinically. Each of the groups was further divided according to age, with a cutoff point of 50 years (NR < 50 = 13, NR ≥ 50 = 16, R < 50 = 32, and R ≥ 50 = 49). The involvement of the PI3K/Akt/mTOR pathway, as well as that of HIF-1*α* and HIF-2*α* and of CD4+, CD8+, and CD19+ cells and mastocytes, was assessed. Saphenous vein tissue samples obtained during surgery were processed for RT-qPCR and immunohistochemistry. Patients with venous reflux showed a significant increase in mRNA and protein expression levels for PI3K/mTOR and HIF-1*α*/HIF-2*α*. The number of mast cells was significantly elevated in the R group. In distribution by age, PI3K/Akt/mTOR and HIF-1*α* were significantly higher in R < 50 patients. Furthermore, these patients had a significant increase in the number of CD4+, CD8+, and CD19+ cells and mastocytes in the saphenous vein wall. These findings provide a basis for the possible existence of changes in PI3K/Akt/mTOR pathway expression in young patients, with potential accelerated asynchronous aging that is enhanced by CVI.

## 1. Introduction

Venous pathology develops when venous pressure increases and blood return is disrupted. The peripheral venous system functions as a conduit that returns blood to the heart. Proper functioning of this system depends on the venous valves and the muscular pump [1]. Chronic venous insufficiency (CVI) is a multifactorial disease, commonly caused by valvular incompetence (clinically visible due to the presence of venous reflux) and venous hypertension [2]. The incidence of varicose veins per year according to the Framingham study is 2.6% in women and 1.9% in men [3]. The incidence clearly increases with patient age, family history of CVI, and history of multiple pregnancies [4]. Aging is considered one of the risk factors involved in this pathology [5]. Pocock et al. [6] reported that cellular events have a special importance in the pathophysiology of CVI and are a starting point for the response and progression of this pathology.

PI3K/Akt/mTOR pathway activity is reported by numerous researchers to be fundamental in vascular pathologies [7, 8]. Classically, PI3K activity has been considered a hallmark of the oncogenic process [9]. Parallel to research by cancer biologists, research in other fields has uncovered disturbing and often unpredictable roles of PI3K in normal cell function and disease [10, 11]. Many of these functions affect cellular homeostasis by influencing cellular dynamics [12]. PI3K induces the phosphorylation of secondary proteins, triggering the recruitment of cytoplasmic proteins [13]. This event activates the cellular PI3K/Akt/mTOR signaling pathway, which is an important intracellular signaling pathway in cell cycle regulation. Therefore, it is directly related to cellular quiescence, proliferation, cancer, and longevity [14, 15]. Balancing the appropriate amount of proliferation versus differentiation is challenging and motivates the need for research to determine this balance for use in the development of different therapies [16].

Varicose veins develop through a series of gradual stages; it is possible to consider the development of venous wall insufficiency as an aging process. Generally, this condition proceeds according to the age of the individual, especially after the fifth decade of life [17]. With aging comes changes in the structure of the venous wall induced by alterations in the connective tissue [18]. This situation is related to loss or poor organization of elastic fibers and muscles of the middle layer, which facilitates venous dilation [17–19]. CVI has been demonstrated in young patients and can be considered a premature and accelerated asynchronous aging process [6, 18, 20]. These young CVI patients may possess a specific genetic background, but no studies on valvular incompetence have been reported. Therefore, in young people with CVI or with certain genetic susceptibility, valvular incompetence becomes an important risk factor. Considering previous research, the PI3K/Akt/mTOR pathway can affect cellular function. Therefore, the aim of this study is to demonstrate how the pathological environment created in the process of valvular incompetence (venous reflux) development in patients with CVI is produced by a set of cellular-level events modulated by the PI3K/Akt/mTOR pathway.

## 2. Patients and Methods

### 2.1. Design of the Study

This is an observational, analytical, and prospective cohort study that reviewed patients with CVI scheduled to undergo stratified saphenectomy and divided them according to age (cutoff point of 50 years). The research was developed through a collaboration between the Service of Angiology and Vascular Surgery of the Ruber International Hospital and the Department of Medicine and Medical Specialties of the University of Alcalá. The study cohort was selected according to the following criteria. Inclusion criteria are as follows: women and men diagnosed with CVI, with and without venous reflux in the great saphenous vein; BMI ≤ 25; informed consent signed; and commitment to have a follow-up during the pre- and postoperative periods plus tissue sample collection. Exclusion criteria are as follows: patients with venous malformations or arterial insufficiency, patients who did not provide their clinical history, patients with pathology affecting the cardiovascular system (infectious diseases, diabetes, dyslipidemia, and hypertension), patients with toxic habits, and patients who doubted they could complete the full follow-up.

Each patient underwent exploration with the aid of an M-Turbo Eco-Doppler (SonoSite) transducer at 7.5 MHz. The examination of the lower limbs was performed in a standing position with the leg that was explored maintained in external rotation and supported by the contralateral leg; the study included the great saphenous axis from the inguinal region to the ankle and femoral vein. A study of the small saphenous vein and popliteal vein was also performed standing, with the back to the examiner and the body weight resting on the examined leg. A distal compression maneuver was performed. In this study, Valsalva maneuvers were performed, which when producing a proximal circulatory stop will allow exploration of venous insufficiency proximal to the detection point, as well as the identification of leakage points (it evaluates the absence of reflux in the femoral-iliac and saphenous-femoral union). The distal compression and decompression maneuver was also performed to assess the direction of the truncal venous flow, although it was not a physiological maneuver. Pathological reflux was considered when this was greater than or equal to 0.5 sec.

The present study was conducted in accordance with the basic ethical principles (autonomy, beneficence, nonmaleficence, and distributive justice), and its development followed Good Clinical Practice standards and the principles set forth in the last Declaration of Helsinki (2013) and the Convention of Oviedo (1997). The patients were duly informed, and each was asked to provide written informed consent. The project was approved by the Clinical Research Ethics Committee of the Ruber International Hospital.

### 2.2. Samples

Once saphenectomy was performed, the entirety of the great saphenous vein was extracted. These fragments were introduced into two different sterile tubes, one containing MEM (minimum essential medium) with 1% antibiotic/antimycotic (both from Thermo Fisher Scientific, Waltham, MA, USA) and another containing RNAlater® Solution (Ambion, Austin, TX, USA). All samples are transferred refrigerated to the Department of Medicine and Medical Specialties (Faculty of Medicine and Health Sciences, University of Alcalá) for processing. In all cases, the transfer was made within four hours after the sample was taken.

### 2.3. Structural Studies

The samples were processed in a Telstar AV 30/70 Müller class II laminar flow hood 220 V 50 MHz (Telstar SA Group, Terrassa, Spain), thus allowing an environment of sterility. Samples conserved in RNAlater remained in 1 mL of the same solution at −80°C until further processing for analysis of gene expression. The samples conserved in MEM were destined for histological studies of venous tissue. The samples were washed/hydrated several times with MEM without antibiotic to remove blood cells and then cut into fragments that were kept in different fixatives: F13 (60% ethanol, 20% methanol, 7% polyethylene glycol, and 13% distilled H_2_O). After the necessary fixing time for each fixative solution, the samples were dehydrated. At the end of the inclusion, paraffin blocks were made using molds. Once the paraffin solidified, an HM 350 S rotation microtome (Thermo Fisher Scientific) was used to obtain 5 *μ*m thick sections on glass slides impregnated with 10% poly-L-lysine solution. Once dry, the sections were deparaffinized for 30 minutes in xylol (PanReac AppliChem, Barcelona, Spain) and then rehydrated by passing through solutions with decreasing alcohol concentrations. After rehydration, the sample sections were subjected to different staining (toluidine blue) and immunohistological processes.

Toluidine blue staining was conducted as follows: (1) staining with toluidine blue in 0.03% aqueous solution for 15 minutes, (2) washing with running water for 10 minutes, (3) dehydration in 96% alcohol for 5 minutes, (4) dehydration in 100% alcohol for 5 minutes, (5) clarification in xylol for 5 minutes, and (6) assembly with Cytoseal™ balsam. Toluidine blue is often used to identify mast cells, by virtue of the heparin present in their granules.

### 2.4. Immunohistochemical Studies

Antigen-antibody detection was achieved using the ABC (avidin-biotin complex) method with chromogenic peroxidase or alkaline phosphatase according to the following protocol: (1) washing the samples with PBS 1x, three passes of 5 minutes. (2) Blocking of nonspecific binding sites with BSA (bovine serum albumin) at 3% in PBS for 30 minutes at room temperature. (3) Incubation with the primary antibody (Table 1) diluted in 3% BSA and PBS overnight at 4°C. (4) Washing with PBS, three passes of 5 minutes each. (5) Incubation with the secondary antibody bound to biotin (Table 2) and diluted in PBS for 1 hour and 30 minutes at room temperature. (6) Washing with PBS, three passes of 5 minutes. (7) Incubation with the avidin-peroxidase ExtrAvidin®-Peroxidase (Sigma-Aldrich, St. Louis, MO, USA) for 1 hour at room temperature. Dilution 1/200 in PBS. In the case that the conjugate was avidin-alkaline phosphatase (ExtrAvidin-Alkaline Phosphatase, Sigma-Aldrich), it was used for 60 minutes at room temperature. Dilution 1/200 in PBS. (8) Washing in PBS, three passes of 5 minutes each. (9) (A) Development by incubation with the chromogenic substrate diaminobenzidine (Kit DAB, SK-4100) (Vector, Burlingame, CA, USA). The preparation of the chromogenic substrate is carried out immediately before development (5 mL of distilled water, 2 drops of buffer, 4 drops of DAB, and 2 drops of hydrogen peroxide). This technique results in labeling with a brown color. (B) Development with the alkaline chromogenic substrate for 15 minutes (controlling the appearance of marking under the microscope). The chromogenic substrate preparation was performed immediately before development by adding 10 mL of PBS (10 mg of *α*-naphthol AS-BI phosphate, 10 mg of Fast Red, and 100 *μ*L of 0.1 M levamisole). (10) Washing with distilled water to stop the development reaction, with three 5-minute passes. (11) Contrasting the nuclei by staining with Carazzi's hematoxylin for 5–15 minutes. (12) Washing in running water for 10 minutes. (13) Mounting in aqueous medium with Plasdone. In all immunohistochemical studies, sections of the same tissue were used as a negative control, in which the incubation with the primary antibody was replaced by incubation in blocking solution.

### 2.5. Genetic Expression

Through real-time polymerase chain reaction (qPCR), the amount of cDNA in each sample of the gene of interest was quantified (Table 3). The results were normalized using the constitutive expression gene of GAPDH (Table 3). Primer-specific primers were designed for all genes studied using the Primer-BLAST [21] and AutoDimer [22] online applications. The extraction of RNA was carried out capped by the guanidine-phenol-chloroform isothiocyanate method of Chomczynski and Sacchi [23]. The qPCR was performed on a StepOnePlus™ System (Applied Biosystems-Life Technologies), using the relative standard curve method. To this end, 5 *μ*L of each sample, previously diluted 1/20 in nuclease-free water, was mixed with 10 *μ*L of iQ™ SYBR® Green Supermix (Bio-Rad Laboratories), 1 *μ*L forward primer, 1 *μ*L Μl of reverse primer, and 3 *μ*L of DNase and RNase-free water in a MicroAmp® 96-well plate (Applied Biosystems-Life Technologies), for a total reaction volume of 20 *μ*L. Fluorescence detection is performed at the end of each repeat (amplification) cycle and at each step of the dissociation curve. The data obtained from each gene are interpolated in a standard curve made by serial dilutions of a mixture of the study samples which is included in each plate. All tests are performed in duplicate.

### 2.6. Statistical Analysis and Evaluation of Expression

For statistical analysis, the GraphPad Prism® 6.0 program was used to apply the Mann–Whitney *U* test. The data are expressed as the mean ± standard deviation of the population. Significance was established at ^∗^*p* < 0.05, ^∗∗^*p* < 0.005, and ^∗∗∗^*p* < 0.001. In the case that the study variables were not qualitative, a Pearson chi-square test or Fisher's exact test was used when applicable. In the case of inequality, possible confounding factors were defined for which the final analysis of the main efficacy variable was adjusted.

For each of the patients in the established groups, five sections and 10 fields per section were randomly selected and examined. The patients were described as positive when the average of the analysis of the labeled sample for each study subject was greater than or equal to 5% of the total, following the anatomopathological protocol of Ortega et al. [24]. Infiltrated cells were counted under a microscope (1000x) in 10 aleatory areas of 0.5 mm^2^ per patient. All values are expressed as means ± SE. Sample observation was carried out using a Zeiss Axiophot optical microscope (Carl Zeiss, Germany) equipped with an AxioCam HRc digital camera (Carl Zeiss, Germany).

## 3. Results

### 3.1. Clinical and Demographic Characteristics

For the present study, a total of 110 patients were contacted. These patients were classified according to the absence of venous reflux (NR; *n* = 29) (51.51 ± 14.04) or the presence of reflux (R; *n* = 81) (50.09 ± 15.91). The classification according to age was established as follows: patients under 50 years of age with an absence of venous reflux (NR < 50; *n* = 13) (38.53 ± 6.21), patients greater than or equal to 50 years with an absence of venous reflux (NR ≥ 50; *n* = 16) (62.06 ± 8.54), patients younger than 50 years with the presence of venous reflux (R < 50; *n* = 32) (35.09 ± 7.31), and patients greater or equal to 50 years with the presence of venous reflux (R ≥ 50; *n* = 49) (59.98 ± 11.81).

There were no significant differences in the hemogram or in the general biochemistry (data not shown). This nonsignificant relationship was maintained when the absence or presence of venous reflux was considered, as well as when age was considered. No significant differences were found in the clinical history of the patients when studying demographic factors (data not shown).

### 3.2. Expression of the PI3K/Akt/mTOR Pathway

The expression of the PI3K/Akt/mTOR pathway was revealed using protein and relative quantity mRNA detection techniques.

#### 3.2.1. PI3K

**Figure 1:**
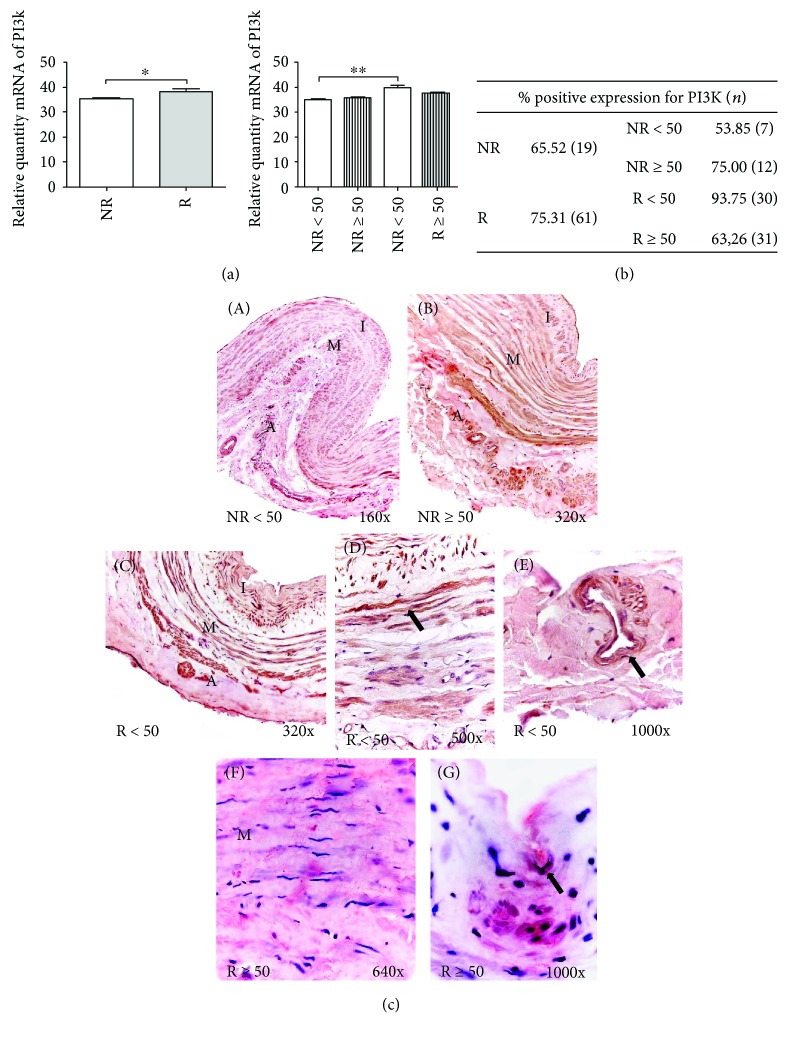
(a) Significant levels of mRNA for PI3K quantified by RT-qPCR in R and R < 50 patients. Results were normalized to that of the reference gene GAPDH and are provided in arbitrary units. NR = no reflux; R = reflux. ^∗^*p* < 0.05 and ^∗∗^*p* < 0.005. (b) Distribution of the percentage of patients with positive protein expression for PI3K in NR and R patients and by age. *n* = number of patients. (c) A-B: Histological images for PI3K protein expression in the different tunicae of venous wall in NR < 50 (160x) and NR ≥ 50 patients (320x). C-D: Images of PI3K expression of R > 50 throughout the vein wall (C, 320x) smooth muscle bundles (arrow) in the tunica media (D, 500x) and in the venula (arrow) of the tunica adventitia (E, 1000x). F-G: PI3K protein expression images in R ≥ 50 patients along the wall of the vein (F, 640x) and in the insertion areas of the venous valves (G, 1000x). The red coloration indicates the specific precipitate that correlates with the expression of the said protein. A = tunica adventitia; M = tunica media; I = tunica intima.

The relative quantity mRNA of PI3K showed a significant increase in patients with R in comparison with the NR group (^∗^*p* < 0.05). In distribution by age, statistically significant differences were established between NR < 50 and R < 50 patients (^∗∗^*p* < 0.005) (Figure 1(a)).

The percentage of patients with positive protein expression based on immunohistochemical studies of PI3K expression was higher (75.31%) in the group of R individuals. Notably, NR ≥ 50 patients and R < 50 patients exhibited greater expression than the rest of the individuals, with 75.00% and 93.75%, respectively (Figure 1(b)). PI3K expression was distributed in the vein wall and was present throughout the NR < 50 and NR ≥ 50 patients (Figure 1(c), A and B).

Patients with venous reflux exhibited differential expression depending on their age. In the R < 50 patients, PI3K expression was observed throughout the entire vein wall (Figure 1(c), C) and was most intense in the smooth muscle bundles of the tunica media (Figure 1(c), D arrow) and in the venula of the tunica adventitia (Figure 1(c), E arrow). In the case of the R ≥ 50 patients, lower PI3K expression intensity was observed. The expression in this group of patients presented as small heterogeneous accumulations along the wall of the vein (Figure 1(c), F), which were especially intense in the insertion areas of the venous valves (Figure 1(c), G arrow).

#### 3.2.2. Akt

**Figure 2:**
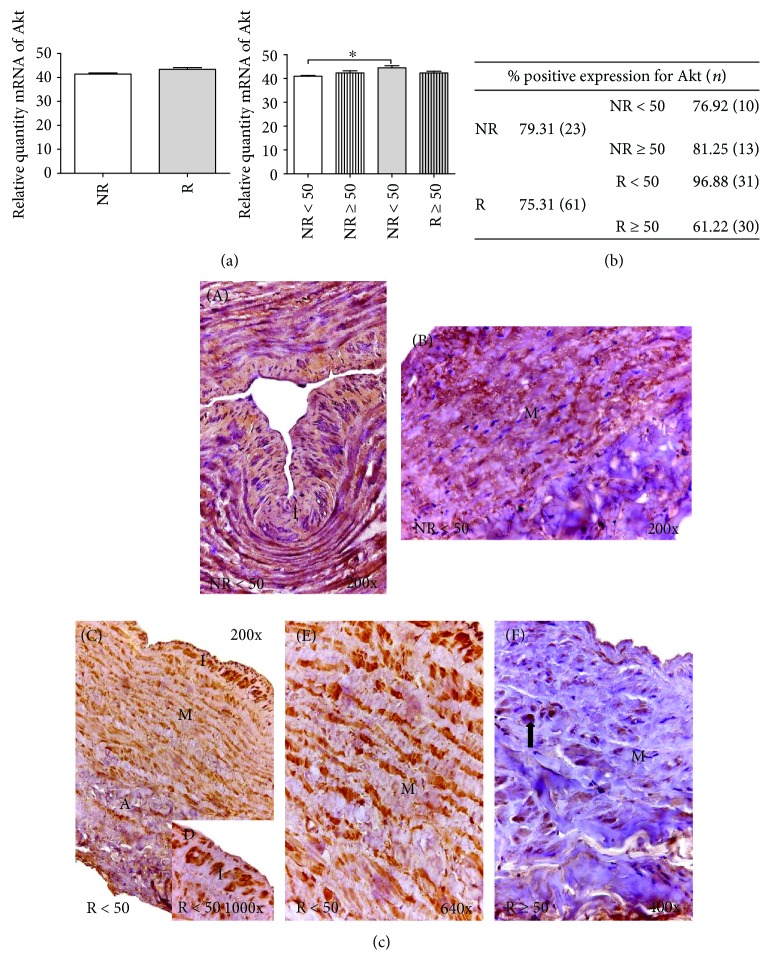
(a) Significant levels of mRNA for Akt quantified by RT-qPCR. In R < 50 patients, results were normalized to that of the reference gene GAPDH and are provided in arbitrary units. NR = no reflux; R = reflux. ^∗^*p* < 0.05. (b) Distribution of the percentage of patients with positive protein expression for Akt in NR and R patients and by age; *n* = number of patients. (c) A–C: Histological images for Akt protein expression in the different tunicae of venous wall in NR < 50 (200x), NR ≥ 50 (200x), and R < 50 patients (200x). D-E: Detail of expression for Akt at greater magnification in R < 50 for tunica intima (640x) and tunica media (640x). F: R ≥ 50 patients show a heterogeneously Akt protein expression as small accumulations (arrow) in the tunica media (400x). A = tunica adventitia; M = tunica media; I = tunica intima. The brown coloration indicates the specific precipitate that correlates with the expression of the said protein.

mRNA expression did not reveal any significant differences between the study groups (NR versus R). The distribution by age showed a significant increase in R < 50 patients (^∗^*p* < 0.05) (Figure 2(a)).

The percentage of Akt protein expression was not different between NR (79.31%) and R (75.31%) individuals. Remarkably, R < 50 patients had the highest percentage of protein expression at 96.88% (Figure 2(b)). In the NR < 50 patients, Akt expression was distributed by the three tunicae of the venous wall (Figure 2(c), A). In the case of the NR ≥ 50 patients, the percentage of individuals with positive expression was greater than that of young patients; however, the distribution was more heterogeneous. Akt was observed in large accumulations along the wall of the vein (Figure 2(c), B).

Microscopic observation revealed that the R < 50 patients had high Akt expression intensity throughout the entire venous wall (Figure 2(c), C). This expression was visualized acutely in the tunica intima (Figure 2(c), D) and measured in the smooth muscle bundles (Figure 2(c), E). The R ≥ 50 patients had low Akt expression. Akt was observed heterogeneously as small accumulations in the tunica media within the smooth muscle fibers (Figure 2(c), F).

#### 3.2.3. mTOR

**Figure 3:**
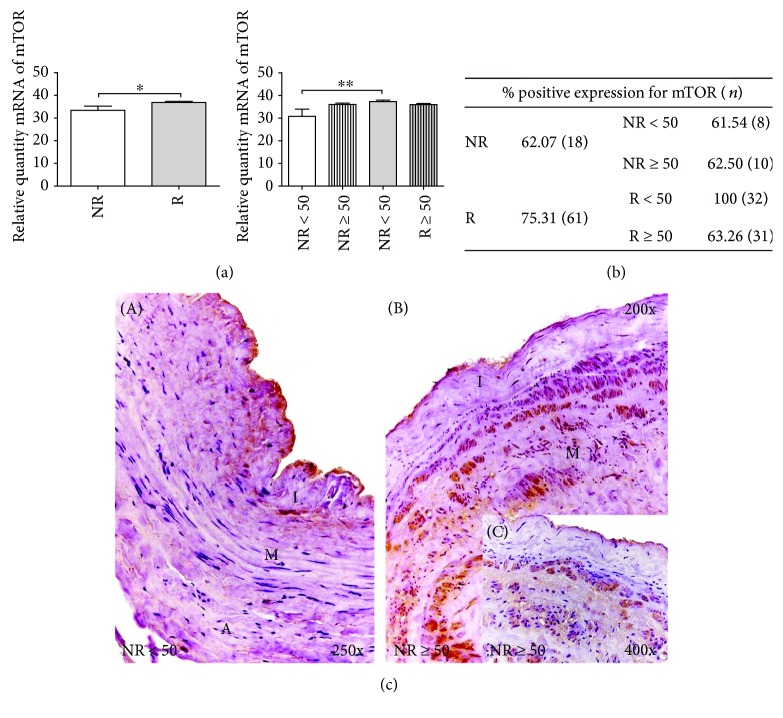
(a) Significant levels of mRNA for mTOR quantified by RT-qPCR in R and R < 50 patients. Results were normalized to that of the reference gene GAPDH and are provided in arbitrary units. NR = no reflux; R=reflux. ^∗^*p* < 0.05 and ^∗∗^*p* < 0.005. (b) Distribution of the percentage of patients with positive protein expression for mTOR in NR and R patients and by age; *n* = number of patients. (c) A: Histological images for protein expression mTOR in the different tunicae of venous wall in NR < 50 patients (350x). B, C: mTOR protein expression images in NR ≥ 50 patients (200x–400x). The brown coloration indicates the specific precipitate that correlates with the expression of the said protein. A = tunica adventitia; M = tunica media; I = tunica intima.

A relative amount mRNA of mTOR was higher in the R patients versus the NR group, establishing a statistically significant difference (^∗^*p* < 0.05). In the case of the distribution by age, the R < 50 patients showed a significant increase in comparison with the NR<50 group (^∗∗^*p* < 0.005) (Figure 3(a)).

The percentage of positive mTOR expression was higher in patients with venous reflux (R), with 62.07% in the NR and 77.78% in the R patients. Consideration of the age factor showed that the percentage was more elevated in the R < 50 patients, being 100% in these patients and 63.27% in the R ≥ 50 patients. In the case of the NR patients, the expression percentages were similar: 61.54 in NR < 50 and 62.50 in NR ≥ 50 (Figure 3(b)).

The microscopic observation showed that mTOR was distributed differently in the different study groups. The NR < 50 patients exhibited reduced mTOR expression, which was limited to the environment of the tunica media and appeared as small heterogeneous accumulations along the wall of the vein (Figure 3(c), A). In the case of the NR ≥ 50 patients, mTOR was present in the tunica media and slightly in the tunica intima (Figure 3(c), B). This expression was irregular, with intense small points in the smooth muscle bundles of the vein wall (Figure 3(c), C).

**Figure 4:**
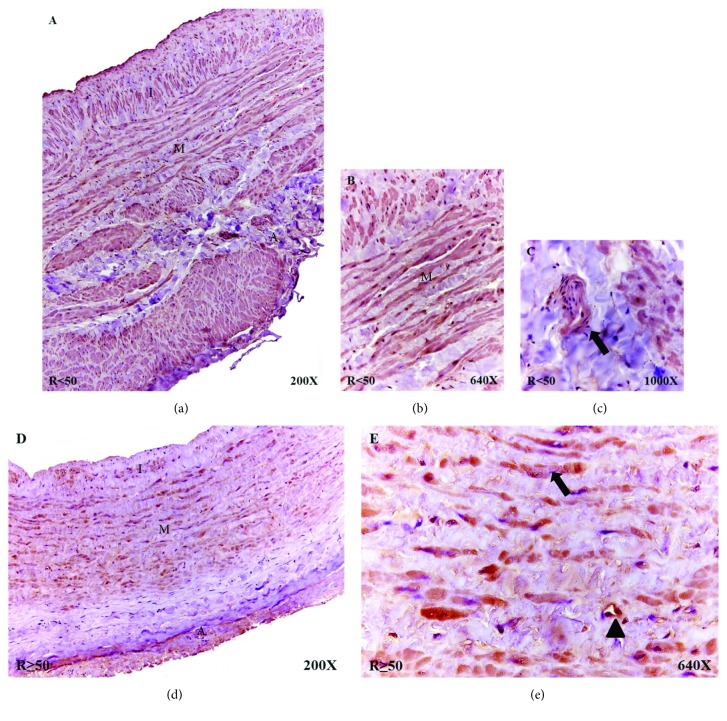
(a) Histological images for protein expression of mTOR in the different tunicae of the venous wall in R < 50 patients (200x). (b-c) Detail of expression for mTOR at greater magnification in media tunica (640x) and venula (arrow) in adventitia tunica (1000x). (d-e) Protein expression images of mTOR in R ≥ 50 patients, with detail in smooth muscle bundles (arrow) and blood capillary (arrowhead) in the tunica media (640x). The brown coloration indicates the specific precipitate that correlates with the expression of the said protein. A = tunica adventitia; M = tunica media; I = tunica intima.

The R < 50 patients had high mTOR protein expression that extended throughout the entire wall of the vein (Figure 4(a)). Importantly, mTOR was differentially distributed in the smooth muscular bundles of the tunica media, with intense labeling observed (Figure 4(b)). In these patients, venula in the tunica adventitia were visualized with a high expression for mTOR protein (Figure 4(c), arrow). The R ≥ 50 patient samples exhibited lower mTOR protein expression intensity. The protein extended along the tunica media and was mildly expressed in the tunica adventitia, with low expression in the endothelium (Figure 4(d)). The smooth muscular bundles appeared labeled (Figure 4(e), arrow) and blood capillary (Figure 4(e), arrowhead), and this expression was heterogeneous.

### 3.3. Expression of HIF-1*α* and HIF-2*α*

The study of the hypoxic component was performed by relative quantity mRNA and protein detection of hypoxia-inducible factors (HIFs) based on the expression of the subunits 1 alpha (HIF-1*α*) and 2 alpha (HIF-2*α*).

#### 3.3.1. HIF-1*α*

**Figure 5:**
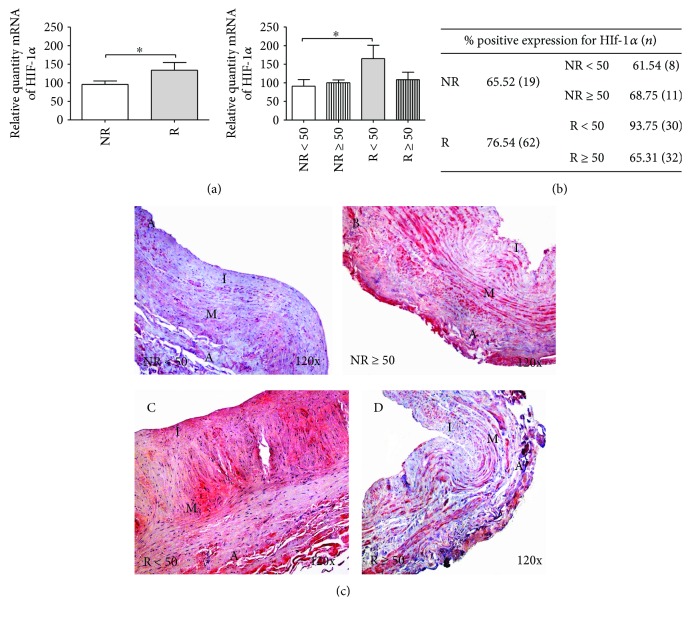
(a) Significant levels of mRNA for HIF-1*α* quantified by RT-qPCR in R and R < 50 patients. Results were normalized to that of the reference gene GAPDH and are provided in arbitrary units. NR = no reflux; R = reflux. ^∗^*p* < 0.05. (b) Distribution of the percentage of patients with positive protein expression in HIF-1*α* NR and R patients and by age; *n* = number of patients. (c) A–D: Histological images for HIF-1*α* protein expression in the different tunicae of the venous wall in NR < 50, NR ≥ 50, R > 50, and R ≥ 50 patients (120x). Red coloration indicates the specific precipitate that correlates with the expression of said protein. A = tunica adventitia; M = tunica media; I = tunica intima.

A relative amount of mRNA of HIF-1*α* was higher in the R group versus the R patients, establishing a statistically significant difference (^∗^*p* < 0.05). The distribution of amount by age showed statistically significant differences between the NR < 50 and R < 50 patients (^∗^*p* < 0.05) (Figure 5(a)).

When studying HIF-1*α* protein expression, differences were observed in the percentage of expression in patients with positive expression. Overall, the R patients presented an expression percentage of 76.54%, but in the R < 50 group, the expression percentage was 93.75%, which was greater than that of patients without reflux (Figure 5(b)). The HIF-1*α* protein expression intensity was similar in all study groups, and a uniform distribution along the vein wall was observed (Figure 5(c)).

#### 3.3.2. HIF-2*α*

**Figure 6:**
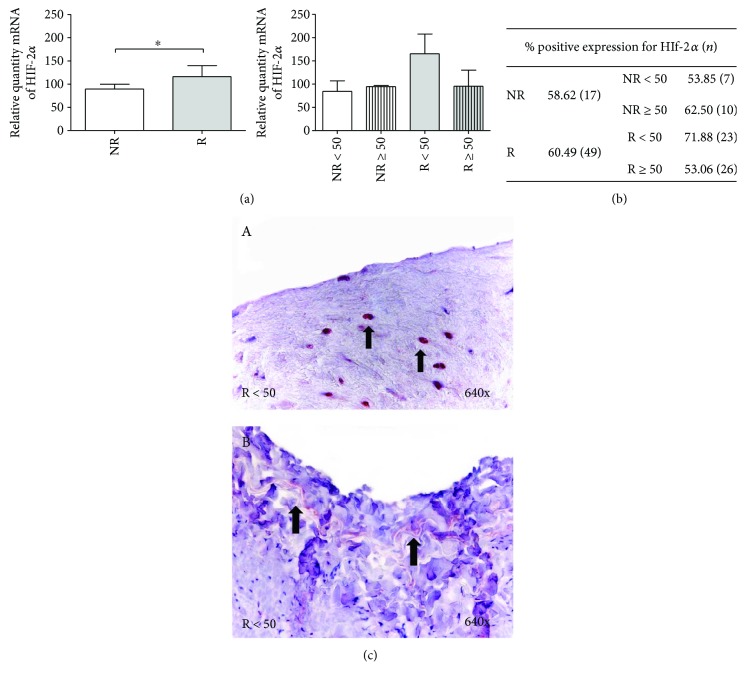
(a) Significant levels of mRNA for HIF-2*α* quantified by RT-qPCR in R. Results were normalized to that of the reference gene GAPDH and are provided in arbitrary units. NR = no reflux; R = reflux. ^∗^*p* < 0.05. (b) Distribution of the percentage of patients with positive protein expression in HIF-2*α* NR and R patients and by age; *n* = number of patients. (c) A: Protein expression images of HIF-2*α* in cell nucleus (arrow) of R > 50 patients (640x). B: Protein expression images of HIF-2*α* in capillary (arrow) of adventitia tunica in R > 50 patients (640x). Red coloration indicates the specific precipitate that correlates with the expression of the said protein.

A relative quantity of mRNA of HIF-2*α* was higher in the R patients versus the NR group, establishing a statistically significant difference (^∗^*p* < 0.05). In the case of distribution by age, mRNA expression did not reveal any significant differences between the study groups (Figure 6(a)).

The study of HIF-2*α* protein expression showed that the percentage of patients reactive for this factor was similar in the NR (58.62%) and R (60.49%) patients. The highest percentage of positive HIF-2*α* expression was observed in the R < 50 patients (71.88%) (Figure 6(b)).

Protein expression was present in the nuclei of smooth muscle fibers (Figure 6(c), A–C); in the R < 50 patients, there was slight expression in the vessels of the tunica adventitia (Figure 6(c), arrow). The rest of the patients presented with diffuse protein labeling similar to that previously described for muscle fibers.

### 3.4. Expression of CD4+, CD8+, and CD19+ and Mast Cells

#### 3.4.1. CD4+ Cells

**Figure 7:**
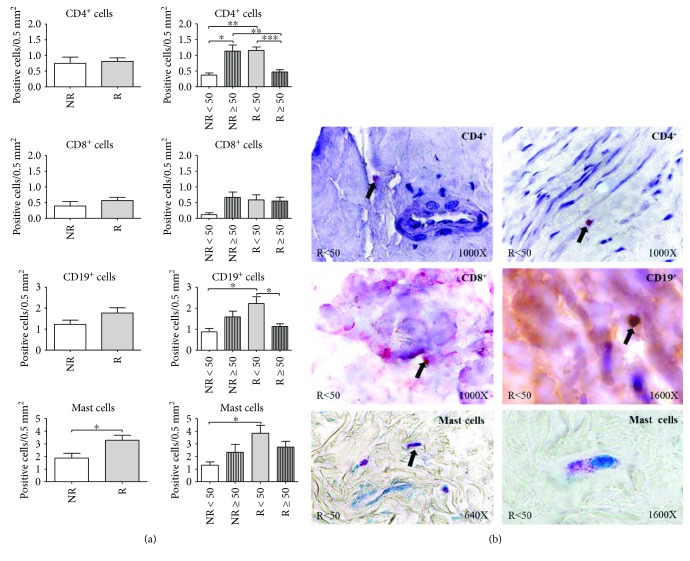
(a) Positive cell quantification of CD4+, CD8+, CD19+, and mast cells in the three tunicae of the vein wall for patients without reflux (NR) and with reflux (R), as well as by the ages of the same. ^∗^*p* < 0.05; ^∗∗^*p* < 0.005; ^∗∗∗^*p* < 0.001. (b) Histological images showing the specific detection of immune cells CD4+, CD8+, and CD19+ and mast cells in R < 50 patients.

In terms of quantification between the NR and R patient groups, no significant differences were observed. By considering age, we found significant differences. The highest number of CD4*+* cells was observed in the NR ≥ 50 and R < 50 patients, which presented significant differences in comparison with the rest of the study groups (Figure 7(a)). CD4+ cells were present along the entire wall of the vein in all the study groups (Figure 7(b)).

#### 3.4.2. CD8+ Cells

Quantification of the number of CD8+ cells did not show significant differences between the NR and R patients. Including age in the analysis did not reveal differences between the different patient groups but showed a slight tendency toward an increase similar to that observed in the CD4+ cells (Figure 7(a)). The CD8+ cells were visualized by immunodetection techniques and appeared in the tunica intima and adventitia of the vein (Figure 7(b)).

#### 3.4.3. CD19+ Cells

The presence of B lymphocytes was evidenced by immunodetection of CD19+ cells, and an increasing tendency in the R patients with respect to the NR patients was found. In terms of age, statistically significant differences were established between the NR < 50 and R < 50 patients (^∗^*p* < 0.05) and between the R < 50 and R ≥ 50 patients (^∗^*p* < 0.05). The data obtained show that the highest elevation of CD19+ cells occurred in the R < 50 patients (Figure 7(a)). CD19+ cells were visualized on the endothelium and tunica adventitia of the vein wall (Figure 7(b)).

#### 3.4.4. Mast Cells

The presence of mast cells in the vein wall was detected with toluidine blue. The results revealed a significant increase in the number of mastocytes in the R patients with respect to the NR patients (^∗^*p* < 0.05). Statistically significant differences were observed between the NR < 50 and R < 50 patients (^∗^*p* < 0.05) (Figure 7(a)). Toluidine blue allowed us to identify these cells because the stain lends them a purple tone due to the mast cell cytoplasmic granules, which are rich in anionic substances, such as histamine and heparin. When performing the microscopy study, mast cells were observed near blood capillaries in the tunica media and adventitia, as well as in the vasa vasorum of the vein wall (Figure 7(b)).

## 4. Discussion

Despite scientific and technological advances, the failure of the venous wall responsible for CVI still has no clear etiology. Therefore, multiple factors have been proposed to contribute to venous wall failure and to potentially determine the failure either by distension of the wall, valvular failure, or valvular agenesis in some key places [25, 26]. The measure of venous reflux is clinically valuable, and one of the tests aids in estimating the degree of venous involvement in the lower limbs. However, to date, there are no clear data on the correlation between reflux and damage to the venous wall, thus limiting the implementation of specific corrective measures.

Previous works have shown that of the multiple noxae that affect the wall and venous functioning, the very process of life (aging) is one contributor to venous failure [18]. CVI with or without reflux will produce a dilatation of the venous wall that initially leads to alterations in the structure of the compensatory wall in the form of hypertrophic areas, and after failure, the wall will return to being fibrosclerotic [27]. These alterations, which are induced, promoted, and maintained by the phenomena of inflammation and ischemia, lead to cytoarchitectonic remodeling of the venous wall, causing in turn a manifest functional incompetence known as venous reflux. Although aging and CVI develop in parallel, the aging process can be accelerated in CVI, coinciding with the secondary remodeling induced by valvular incompetence [27–29]. CVI induces changes in the return of venous flow, which can increase venous filling and subsequently induce an increase in intraluminal pressure. This would produce an increase in venous stasis and relative hypoxia, with a consequent associated increase in oxidative metabolism and reactive oxygen species. All this would contribute to venous wall remodeling and damage [30].

One of the events observed by our research group is the gene and protein expression of different components of the PI3K/Akt/mTOR cellular transduction pathway. Our results have shown that patients with venous reflux, especially young individuals, have a significant increase in vein activation. Yuan et al. [31] noted that PI3K is essential in the regulation of embryonic vasculogenesis, and alterations in this pathway can have severe consequences. The implication of these molecules in different pathologies is well tested [32–34], and they are described as triggers for numerous pathophysiological processes. Among these processes, we must highlight hypertension. Another of the points of interest noted by numerous authors is that inflammation processes can trigger the activation of these pathways with severe consequences in the tissue itself [35]. Castel et al. [36] and Castillo et al. [37] reported that mutations in this pathway can have consequences in the vascular system, at both the cytoarchitectural and the physiological levels. Therefore, in light of what was previously stated, the PI3K/Akt/mTOR pathway, as observed by numerous authors, may have an essential role in the remodeling processes in human veins with CVI, especially in those with valvular incompetence.

At another point, hypoxia caused by venous hypertension sets in motion molecular pathways involved in the cellular response to the lack of oxygen, such as PI3K/Akt/mTOR and consequently the HIF transcription factors. Research in the field of these factors suggests that HIF-2*α* stabilization requires less severe hypoxia than HIF-1*α*, but the mechanisms involved are still unknown. Therefore, it is thought that HIF-2*α* should be a first line of response in the face of moderate or less severe decreases in O_2_ [38]. In general, we observed the presence of HIF-1*α* in all the patients studied, which suggests a significant level of hypoxia in the venous walls studied. This hypoxia appeared in the young population with venous reflux at a higher proportion. Related to this event is the mTOR gene and protein expression that has a similar pattern, unlike other PI3K/Akt/mTOR pathway components. In general, our results coincide with those of authors who observed a significant increase in the expression of the HIF pathway in patients with varicose pathology. There has been talk about deregulation of this pathway, which produces an increase in angiogenic factors [39–42]. Lee et al. [39, 40] described a significant increase in HIF-1*α* in the muscle layers of diseased vessels. They observed that this expression was related to the increased Bcl-2 in the vessel endothelium, which would lead to apoptosis inhibition and therefore to an increase in the dilation of the wall of the human vein.

Interestingly, in our study, we observed that reflux becomes a point of inflection for adaptation relative to the age profile of the patients studied, especially in relation to mTOR expression. All these findings are compatible with the process of the hypertrophy-atrophy sequence that an insufficient venous wall undergoes during this process, which is aggravated in venous reflux due to valve incompetence, as shown by Buján et al. [27].

The presence of CD4+, CD8+, and CD19+ cells suggests the existence of an inflammatory process and stress in relation to the above. A variety of studies have shown an increase in degranulation and extravasation of leukocytes in patients with venous hypertension. Saharay et al. [43] showed via flow cytometry an alteration in the number of neutrophils and monocytes in patients with CVI with venous hypertension. T lymphocytes have been demonstrated to be at least partially involved in the inflammatory process observed in venous disease. By analyzing the blood obtained from varicose veins of patients with CVI, Ojdana et al. [44] were able to demonstrate that the levels of CD4+ T lymphocytes measured from varicose veins were significantly elevated compared with the total CD4+ cell levels. The increase in the level of these cells, as well as other subpopulations of T cells measured by this group, indicate that T cells may also partially participate in the pathogenesis of cardiovascular disease. The increase in the number of CD19+ cells in patients with venous reflux could be related to changes in the inflammatory status produced by the increase in CD4+ cells. Zhang et al. [45] proposed that the presence of CD19+ cells was related to PI3K/Akt/mTOR pathway activation, with systemic consequences.

In relation to the above, it should be noted that mast cells can perform a wide variety of functions that induce the inflammatory cascade via mediators derived from their granules in endocytosis. Pascual et al. [18] showed significantly greater mast cell infiltration in varicose vein walls than in healthy controls. Chymase from mast cells is an MMP activator and stimulates the release of TGF-*β*, which plays an integral role in vascular remodeling. Mast cells also secrete tryptase, which can degrade elastin, collagen, proteoglycans, and fibronectin, consequently affecting extracellular matrix remodeling of the vessel wall.

Current research suggests that the PI3K/Akt/mTOR pathway plays an important role in cellular metabolism regulation and in the functions of the immune system, two subjects that are closely related to cell and organ functionality [16, 46]. All this makes us consider the importance of this pathway in the homeostasis of the venous wall and in its activation capacity in pathological processes. Therefore, these facts provide a basis for the possible existence of changes in the PI3K/Akt/mTOR pathway expression in young CVI patients, with a possible accelerated asynchronous aging. In these individuals, the presence of gene mutations or epigenetic changes, as expressed by some authors [47], may play a role. Our results show that older patients with venous reflux have less expression of this pathway. We think that this expression decrease could be explained in relation to cellular senescence, autophagy, and/or apoptosis. In our studied population, patients did not have an oncogenic background. Buján et al. [27] observed that older patients with varicose veins had more TUNEL-positive cells. This fact could be related to that these patients have less activity of PI3K/Akt/mTOR, because they have lost the ability to react to the aggression and they have damage that makes them enter apoptosis. In contrast, young patients with venous reflux have greater expression, which could be related according to some authors [12, 48, 49] with possible cellular senescence and autophagy process. Young patients with venous reflux have a greater ability to react to the cell damage that occurs.

Our results help demonstrate the notion that among several possible mechanisms of activation, venous reflux in CVI is to a considerable degree an inflammatory disease induced by blood pressure. The venous pressure elevation (change in the type of pressure) and the displacement of the shear stress generate an abnormal biomechanical environment in the venules, in their walls, and in the valves, which can initiate early activation of enzymatic activity and in turn set in motion a cascade of cellular transduction, leading to cytoarchitectonic changes to compensate for these alterations.

## Figures and Tables

**Table 1:** Primary antibodies that were used and their dilutions.

Antigen	Species	Clone	Dilution	Provider	Protocol specifications
Akt	Rabbit	Polyclonal	1 : 1000	Abcam (ab8805)	—
CD4	Rabbit	Monoclonal	1 : 50	Abcam (ab133616)	EDTA pH = 9 before incubation with blocking solution
CD8	Rabbit	Polyclonal	1 : 25	Abcam (ab4055)	EDTA pH = 9 before incubation with blocking solution
CD19	Mouse	Monoclonal	1 : 200	Abcam (ab31947)	—
HIF-1*α*	Mouse	Monoclonal	1 : 800	Abcam (ab16066)	EDTA pH = 9 before incubation with blocking solution
HIF-2*α*	Mouse	Monoclonal	1 : 2000	Abcam (ab8365)	EDTA pH = 9 before incubation with blocking solution
PI3K	Mouse	Monoclonal	1 : 500	Abcam (ab86714)	—
mTOR	Rabbit	Polyclonal	1 : 500	Abcam (ab1093)	—

**Table 2:** Secondary antibodies that were used and their dilutions.

Antigen	Species	Clone	Dilution	Provider
IgG (mouse)	Goat	Polyclonal	1/300	Sigma
IgG (rabbit)	Mouse	RG-96	1/1000	Sigma

**Table 3:** Primers used for RT-qPCR: sequences and binding temperatures (Temp).

Gene	Sequence fwd (5′ → 3′)	Sequence rev (5′ → 3′)	Temp (°C)
GAPDH	GGA AGG TGA AGG TCG GAG TCA	GTC ATT GAT GGC AAC AAT ATC CAC T	60
Akt	TGT CTC GTG AGC GCG TGT TTT	CCG TTA TCT TGA TGT GCC CGT C	60
HIF-1*α*	ACG TGT TAT CTG TCG CTT TGA G	ATC GTC TGG CTG CTG TAA TAA TG	59
HIF-2*α*	ACC CAG TAC CAG GAC TAC AGC	GGC ACG TTC ACC TCA CAG TC	61
PI3K	CTT GCC TCC ATT CAC CAC CTC T	GCC TCT AAT CTT CTC CCT CTC CTT C	60
mTOR	ATC CAG ACC CTG ACC CAA AC	TCC ACC CAC TTC CTC ATC TC	60

## Data Availability

The data used to support the findings of this study are available from the corresponding author upon request.
